# Analytical Validation of Familial Hypercholesterolemia Biomarkers in Dried Blood Spots

**DOI:** 10.3390/ijns8010014

**Published:** 2022-02-09

**Authors:** Patrice K. Held, Kristin Campbell, Amy E. Wiberley-Bradford, Michael Lasarev, Vanessa Horner, Amy Peterson

**Affiliations:** 1 Department of Pediatrics, School of Medicine and Public Health, University of Wisconsin, Madison, WI 53706, USA; apeterson@pediatrics.wisc.edu; 2 Wisconsin State Laboratory of Hygiene, School of Medicine and Public Health, University of Wisconsin, Madison, WI 53706, USA; Kristin.Campbell@slh.wisc.edu (K.C.); Amy.Wiberley-Bradford@slh.wisc.edu (A.E.W.-B.); 3 Department of Biostatistics and Medical Informatics, School of Medicine and Public Health, University of Wisconsin, Madison, WI 53706, USA; lasarev@biostat.wisc.edu; 4 Department of Pathology, School of Medicine and Public Health, University of Wisconsin, Madison, WI 53706, USA; Vanessa.Horner@slh.wisc.edu

**Keywords:** newborn screening, familial hypercholesterolemia, cholesterol, low-density lipoprotein, apolipoprotein B, dried blood spots

## Abstract

Heterozygous familial hypercholesterolemia (HeFH) is a common, treatable genetic disorder characterized by premature atherosclerosis and cardiovascular disease, yet the majority of affected individuals remain undiagnosed. Newborn screening could play a role in identification of at-risk individuals and provide an opportunity for early intervention, prior to the onset of symptoms. The objective of this study was to develop and validate assays for quantification of candidate HeFH biomarkers in dried blood spots (DBS). Commercially available enzyme assay kits for quantification of serum total cholesterol (TC) and low-density lipoprotein-cholesterol (LDL-C) were modified for high-throughput analysis of DBS. Apolipoprotein B (ApoB) concentrations in DBS were measured using an immunoassay with modifications from published studies. All three assays were validated according to the College of American Pathologists guidelines for clinical laboratories. The performance of TC, LDL-C, and ApoB assays was assessed by precision, recovery, limit of quantification (LOQ) and linearity. Precision studies yielded coefficients of variation (CV) of less than 15%, with recovery greater than 75% for all three assays. The determined LOQ and linearity were comparable to serum-based assays. In a direct comparison between serum and DBS concentrations, positive correlations were demonstrated for TC, LDL-C, and ApoB. Additionally, the initial evaluation of the three biomarker concentrations within the unaffected population was similar to values obtained in previous published studies. This study reports on methods for quantification of TC, LDL-C, and ApoB in DBS. Assay validation results were within acceptable limits for newborn screening. This is an important first step toward the identification of newborns with HeFH.

## 1. Introduction

Heterozygous familial hypercholesterolemia (HeFH) is the most common, potentially fatal genetic disease in humans, with an incidence of about 1 in 200–300 individuals across all ethnic groups [1]. HeFH is characterized by markedly increased concentrations of low-density lipoprotein-cholesterol (LDL-C) that are present from birth, causing early and aggressive atherosclerotic plaque formation. This predisposes affected individuals to early onset of atherosclerotic cardiovascular disease [1,2,3,4]. In the United States, HeFH is typically diagnosed from a cholesterol blood test, and treatment with statin medications has been shown to be safe, efficacious, and cost effective [1,5,6]. When identified and treated early in childhood, the atherosclerotic plaque formation can be slowed and even reversed, preventing heart disease and early death [7,8,9,10]. Much more rarely, individuals may be affected with the homozygous form of familial hypercholesterolemia (HoFH). HoFH affects an estimated 1 in 160,000 to 400,000 individuals and is characterized by even higher levels of LDL-C, ranging from 500 to 1000 mg/dL. Untreated HoFH can cause ASCVD events as young as the first decade of life, as well as aortic valve disease. Affected individuals rarely live past 30 years old unless heroic measures are undertaken, including treatment with multiple lipid-lowering medications, LDL-C apheresis, and/or organ transplant [11,12].

Despite the widespread availability of LDL-C testing and the effectiveness of treatment, HeFH remains profoundly underdiagnosed, with only 10% of people knowing they have the disease, and of these, only half take cholesterol-lowering medication [1]. Individuals with HoFH are typically identified in adolescence but would benefit from detection and intervention at the youngest age possible [11]. Strategies to improve detection of both HeFH and HoFH in children include the guidelines from the National Heart, Lung, and Blood Institute and the American Academy of Pediatrics advising universal childhood screening between ages 9–11 and again between 17 and 21 years old [1,5,6]. Unfortunately, this recommendation has not significantly increased identification of affected children, due in part to the required logistical steps: a health care visit, a venous blood draw at an age when children frequently resist, and a separate follow-up visit to discuss results. To overcome these barriers, it may be feasible to leverage the existing universal newborn screening programs, which have greater than 98% compliance, to detect children with familial hypercholesterolemia, similar to other inborn errors of metabolism [13].

Initial studies assessing candidate biomarkers (total cholesterol (TC), LDL-C, and apolipoprotein B (ApoB)) in newborns were conducted in cord blood [14,15,16,17,18,19]. In one study, LDL-C was shown to differentiate HeFH cases from controls better than TC [17], while another study concluded that ApoB, not TC or LDL-C, was the superior biomarker [20,21]. To date, no consensus has been reached on the effectiveness of each biomarker. Additionally, for assessment of biomarkers using established newborn screening programs, analytical methods would need to be adapted for the dried blood spot (DBS) specimen. Previous studies measuring TC have used commercially available serum assay kits, based upon a three-step enzymatic reaction, that were modified for DBS [22,23,24]. For ApoB, published methods for quantification in DBS describe the use of radial immunodiffusion [25], immunoturbidity [21,26], or immunoassays [27] with a capture antibody specific for ApoB. However, these published reports have been limited in total specimen numbers or have failed to demonstrate whether these assays could be implemented in the high-throughput screening laboratory (greater than 300 specimens per day, at minimum). To the best of our knowledge, there are no published methods on the quantification of LDL-C in DBS, although it is recognized as the optimal marker for detection of HeFH and HoFH.

The primary goal of our research group is to investigate the feasibility of measuring candidate biomarkers in DBS and to determine whether the existing NBS system could be used to identify individuals with HoFH and HeFH. The first step toward this goal was to develop and validate analytical methods for quantification of TC, LDL-C, and ApoB in DBS that can be easily adapted to the high-throughput setting of a newborn screening laboratory. This publication describes our validation results and provides an initial look at the distribution of biomarker concentrations in newborns.

## 2. Materials and Methods

### 2.1. Specimens

Protocol 2020-0395, approved by the University of Wisconsin Health Sciences Internal Review Board (HS-IRB) on 20 March 2021, allows for use of de-identified, residual newborn screening specimens (DBS), collected between 24–72 h after birth. The concentrations of biomarkers TC, LDL-C, and ApoB were measured in 820 specimens to define the biomarker distribution within the presumptively unaffected population. The specimens were stored at room temperature (RT) for approximately 10–14 days prior to analysis of the biomarkers.

Protocol 2019-0100 was approved by the University of Wisconsin Health Sciences Internal Review Board (HS-IRB) on 24 June 2019. This protocol allowed for the analysis of TC, LDL-C, and ApoB in both serum and DBS obtained from the same venous blood draw of 48 adult volunteer research subjects. The assessment of serum concentrations of TC, LDL-C, and ApoB was performed by the University of Wisconsin Hospitals and Clinics. The protocols used for measurement of serum concentrations were not available to our research group.

### 2.2. Standards and Quality Control Materials

Standards and quality control materials (two levels referenced as control 2 and 3 (ctrl 2, ctrl 3)) for the TC and LDL-C assays were purchased from Point Scientific, Canton, MI, USA (cat# C7509-STD for TC standard, cat# H7545-CAL for LDL-C standard, and cat# L7580-18 for TC and LDL-C controls). The lyophilized powders of standards and controls were resuspended in water following the manufacturer’s recommendation. To prepare DBS specimens, the resuspended standards and controls were mixed 1:1 with packed red blood cells (~50% hematocrit) and spotted onto 903 Whatman filter paper. The specimens were dried overnight and stored at −80 °C. According to the manufacturer’s specification sheet, the concentrations of TC and LDL-C in the individually prepared standards were 200 and 129 mg/dL serum, respectively. For assessment of linearity and limit of quantification (LOQ), concentrated liquid TC and LDL-C standards were serially diluted with hypo-opticlear serum devoid of TC and LDL-C. Each targeted level was mixed 1:1 with packed red blood cells, spotted onto filter paper, dried overnight, and stored at −80 °C until use.

Standards and quality control materials (two levels referenced as control 2 and 3 (ctrl 2, ctrl 3)) for the ApoB assay were purchased from Diazyme, Poway, CA, USA (cat# DZ141A-CAL for standard and cat# DZ248A-CON for the controls). The lyophilized ApoB standard was resuspended in water following the manufacturer’s recommendation (200 mg/dL serum). ApoB calibrators were prepared by serial dilution of the standard into eight levels ranging in concentration from 200 to 2 mg/dL serum. Each calibrator level was mixed 1:1 with packed red blood cells, spotted onto filter paper, dried, and stored at −80 °C until use. These specimens were also used for assessment of linearity and LOQ. ApoB quality control specimens were prepared similarly by mixing purchased multi-analyte lipid control set, reconstituted according to the manufacturer’s recommendation, with packed red blood cells; these were spotted onto filter paper, dried, and stored at −80 °C.

An additional DBS control (Ctrl 1), representing unenriched levels of TC, LDL-C, and ApoB was created by mixing purchased normal human serum in equal amount with packed red blood cells (50% hematocrit), spotted onto filter paper, dried overnight, and stored at −80 °C.

### 2.3. TC and LDL-C Assay in DBS

The first step of the TC and LDL-C assays was the extraction of biomarkers from DBS. For TC, a 3/16” punch was removed from the spotted whole blood and placed into a 96-well non-coated, polypropylene microtiter plate. The LDL-C assay used two, 3/16” punches of each specimen. Methanol (120 µL) was added to each well of the TC and LDL-C plates for extraction of biomarkers and the plate was incubated with shaking at 37 °C for 30 min. Step-wise enzymatic assays to measure TC were performed, with modifications, using the cholesterol liquid reagent kit from Pointe Scientific, Canton, MI, USA (cat# C7510-500). Quantification of LDL-C was performed using the direct LDL-cholesterol kit from Randox, United Kingdom (cat# CH2656).

For TC, 200 µL of proprietary reagent 1 containing cholesterol esterase, oxidase, and peroxidase was added to 25 µL of the methanol DBS extract, followed by incubation for 5 min at 37 °C. The first two steps allowed for the creation of hydrogen peroxide, which in the third step, acted on phenol and 4-aminoantipyrine to produce a red quinoneimine dye that was evaluated spectrophotometrically at 500 nm using a victor3 (Perkin Elmer) spectrophotometer. The absorbance of the specimen, as compared to that of the TC standard, could be used to calculate the amount of TC in the specimen. For LDL-C, an initial step to isolate only LDL particles, consuming HDL, VLDL and chylomicrons, was necessary prior to the three-step enzymatic assessment of cholesterol. For each specimen, 150 µL of proprietary reagent 1 was added to 50 µL of the methanol extract, incubated while shaking at 37 °C for 5 min, followed by assessment of absorbance at 600 nm wavelength using a victor3 (Perkin Elmer) spectrophotometer. Next, 50 µL of reagent 2 was added to each well for the quantification of liberated cholesterol from the LDL-C. The plate was incubated with shaking at 37 °C for 10 min, and the absorbance was measured again at 600 nm. The difference in absorbance between the two steps was compared to the absorbance difference of the LDL-C standard to calculate the concentration of LDL-C. All specimen results for TC and LDL-C are reported in mg/dL serum.

### 2.4. ApoB Assay in DBS

Quantification of ApoB in DBS was performed using a published enzyme-linked immunoassay (ELISA) method with modifications that allowed for high-throughput specimen analysis [27]. Through evaluation of a six-level DBS calibration curve (0–100 mg/dL serum), ApoB concentrations in the specimens were determined. All reagents, including the antisera, were purchased from Mabtech, OH, USA.

High protein-binding capacity 96-well ELISA plates were coated with 100 μL of capture antibody (mAb LDL 20/17) diluted to a final concentration of 2 μg/mL in phosphate buffered saline (PBS) and stored refrigerated overnight prior to use. ApoB in the DBS specimens and controls was extracted using 200 µL of Apo ELISA assay buffer for 1 h and 40 min, shaking at 250 rpm at RT, after which 200 µL of 1% Triton X-100 was added to each sample and the plate was shaken for an additional 20 min. During the extraction, the high protein-binding plate was removed from the refrigerator, washed twice with 400 μL per well of PBS, blocked at RT for 1 h with 400 μL PBS + 0.05% Tween 20 + 0.1% bovine serum albumin (PBS-T + BSA), and then washed again five times with wash buffer (400 μL PBS + 0.05% Tween). A 1:40 dilution of each DBS ApoB extract was prepared in 1× Apo ELISA buffer and 100µL was applied to the coated and blocked high-binding plate. The plates were then sealed and incubated at RT for 2 h, followed by five washes with wash buffer. For detection of the ApoB, 100 µL of the biotinylated detection antibody (mAb LDL 11) at a concentration of 1 µg/mL in 1× Apo ELISA buffer was applied to each well and incubated at RT for 1 h, followed by five washes. Next, 100 µL of streptavidin-horseradish peroxidase (SA-HRP) at a concentration of 1:1000 in PBS-T + BSA was added to each well and incubated for 1 h, followed by five washes. For detection, 100 µL of tetramethylbenzidine substrate was added and the plate was incubated in the dark for 15 min. The reaction was stopped by addition of 100 μL of 2 M H_2_SO_4_. Absorbance at 450 nm was then read using a victor3 spectrophotometer. Specimen ApoB concentrations were determined by comparison to the calibration curve, and the final concentrations were reported in mg/dL serum.

## 3. Results

### 3.1. Analytical Assay Validation of Biomarkers in DBS

The analytical validation of methods to measure TC, LDL-C, and ApoB in DBS included inter-day precision and recovery, limit of linearity, and LOQ. Method precision for TC, LDL-C, and ApoB was assessed through analysis of three quality control materials: endogenous levels (ctrl 1), and two spiked control specimens (ctrl 2 and ctrl 3). The analysis was performed by evaluation of five replicates of each control on five different days (n = 25). The coefficient of variation (CV) for each control, across all three assays, was less than 15% (Table 1).

The recovery of TC from the DBS controls ranged from 76% to 90%, and the recovery of LDL-C was similar, ranging from 74% to 90%. For ApoB, the percent recovery was slightly higher than TC and LDL-C, ranging from 81 to 104% (Table 1).

The limit of linearity for TC, LDL-C and ApoB exceeded the expected thresholds used clinically to distinguish the unaffected from the affected population. Linearity was established for TC and LDL-C up to 900 and 387 mg/dL serum, respectively. For ApoB, the assay was linear to 150 mg/dL serum. The LOQ for each of the three biomarkers was determined by assessment of diluted standards. The concentration at which a percent CV of less than 15 was achieved marked the LOQ. For all three biomarkers, the LOQ was lower than the expected concentrations within the unaffected population (100 mg/dL serum for TC, 40 mg/dL serum for LDL-C, and 2.5 mg/dL serum for ApoB). The limit of detection, determined by the concentration at which an absorbance different from the unenriched dried blood spot could be reliably measured (CV less than 30%), was roughly half of the LOQ for LDL-C and ApoB (Table 2). For TC, however, a true limit of detection could not be achieved because of the endogenous cholesterol (at an estimated ~49 mg/dL serum) present within the red blood cells used to make the DBS calibrators and controls.

### 3.2. Stability

**Figure 1:**
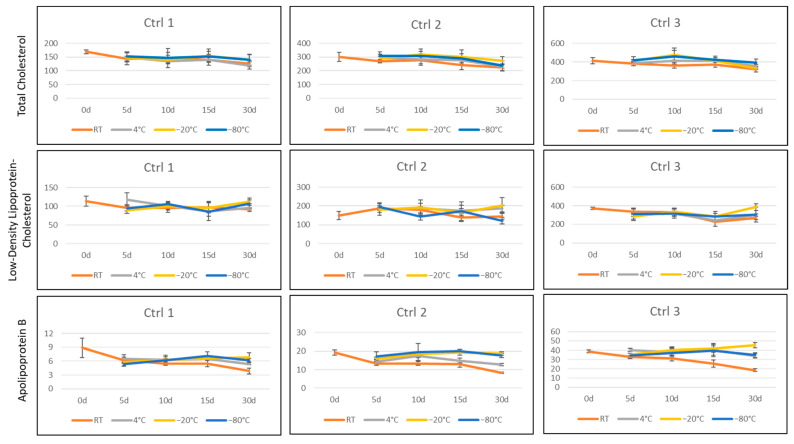
Stability studies. At each time point (0, 5, 10, 15, and 30 days), the three controls (Ctrl 1, 2, and 3) stored at four different temperatures (RT, 4, −20, and −80 °C) were evaluated in 5 replicates. Error bars represent standard deviation among the five replicates.

At five time points (0, 5, 10, 15, and 30 days), three controls (Ctrl 1, 2, and 3) stored at four different temperatures (RT, 4, −20, and −80 °C) were evaluated in five replicates. The concentrations of TC and LDL-C did not appear to vary significantly between storage conditions, nor was the degradation apparent, over the 30 days. However, ApoB controls stored at RT appeared to have significant degradation (loss of 55% from initial value) at 30 days. ApoB controls stored at the three other temperatures degraded, on average, approximately 16% from initial values when evaluated at 30 days (Figure 1). For routine newborn screening, the average time from collection to receipt in the laboratory is less than 7 days. In this analysis, the percent degradation for TC and LDL-C when stored at room temperature for 10 days was minimal (10.5% and 2.1%, respectively). However, ApoB degraded approximately 29% from original evaluation when stored at room temperature for 10 days.

### 3.3. Comparison of Serum and DBS Biomarker Concentrations

**Figure 2:**
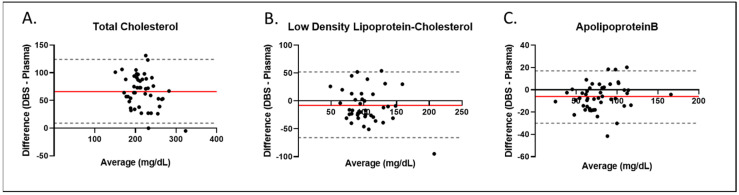
Comparison of Serum to DBS Assays. The Bland–Altman plots show the variation in the serum as compared to the DBS concentration for specimens collected concurrently in 48 research subjects. (**A**) Total cholesterol; (**B**) low-density lipoprotein-cholesterol; (**C**) apolipoprotein B. Red line, bias; dashed Line, 95% limits of agreement.

Serum and DBS were collected concurrently in 48 research subjects, and the concentrations of TC, LDL-C, and ApoB were measured in each specimen type. The Bland–Altman plots in Figure 2 show the variation in the concentrations of serum, as compared to DBS, across the expected range of values. For TC, the DBS values are higher than the serum values by approximately 60 mg/dL. This is likely due to the presence of TC in the red blood cells. For LDL-C and ApoB, the DBS values are slightly lower than the serum values, likely due to inefficiencies in extraction, as observed in the recovery studies.

### 3.4. Quantification of Biomarkers in Presumptively Unaffected Newborns

**Figure 3:**
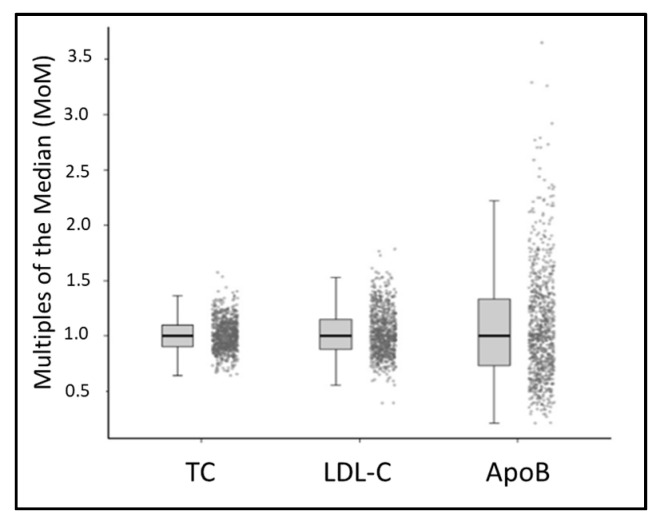
Evaluation of presumptively unaffected population. Multiples of the median for total cholesterol (TC), low-density lipoprotein (LDL-C), and apolipoprotein B (ApoB) in 820 newborn specimens collected within 24–72 h after birth.

De-identified, residual NBS specimens collected between 24–72 h of life from 820 presumptively unaffected newborns were analyzed for all three biomarkers (Figure 3). The TC concentrations showed a Gaussian distribution ranging from 118 to 358 mg/dL, with a mean of 210.2 ± 33.89 mg/dL (median = 208.0 mg/dL; 1st–99th percentile; 140.4–294.6 mg/dL). The distribution for LDL-C was also uniform, with concentrations ranging from 31 to 167 mg/dL, with a mean of 95.79 ± 22.28 mg/dL (median = 93 mg/dL; 1st–99th percentile; 56–153.8 mg/dL). ApoB concentrations exhibited a gamma distribution, with significantly more specimens yielding higher deviations from the mean. The ApoB concentrations ranged from 2.09 to 45.05 mg/dL, with a mean of 12.15 ± 6.329 mg/dL (median = 10.61 mg/dL; 1st–99th percentile; 3.191–32.56 mg/dL). Individual TC, LDL-C, and ApoB values for each specimen were normalized by comparison to other specimens evaluated within the same plate/run using the multiple of the median (MoM) calculation. This analysis minimized the inter-run variation (Figure 3).

## 4. Discussion

The Centers for Disease Control and Prevention’s Office of Public Health Genomics classifies HeFH as a Tier 1 genomic application, indicating significant public health benefit from identifying people at risk. Multiple studies have documented that early treatment of HeFH, beginning as early as 8 years of life, correlates directly with prevention of premature ASCVD and death in adulthood [7,8]. Given the profound benefit of early detection and treatment, clinicians have evaluated multiple strategies for population-wide screening for HeFH, yet most approaches have failed, often due to poor compliance with recommendations. As a result, HeFH remains profoundly underdiagnosed, with greater than 90% of the affected population unaware of their disease state [1].

To the best of our knowledge, newborn screening, a tremendously successful public health program with a compliance rate of greater than 98%, has not been systematically utilized for detection of HeFH. There are both advantages and disadvantages to newborn screening for familial hypercholesterolemia. One advantage of early detection of HeFH in the newborn period is that it would allow for establishment of a heart-healthy lifestyle from the beginning of life, enable appropriate monitoring, and permit initiation of treatment. A critical second advantage is that parents and other relatives could be evaluated for HeFH through cascade screening, providing an opportunity for treatment and prevention in individuals at high-risk for premature morbidity and mortality from cardiovascular disease. Lastly, we anticipate that individuals with HoFH would also be identified. Although HoFH is extremely rare compared to HeFH, the incidence is similar to that of other disorders on the current recommended uniform screening panel [28], and affected individuals would clearly benefit from diagnosis and access to treatment [11,12]. Potential disadvantages of newborn screening for HeFH include added stress, anxiety, or stigma in the first years of life associated with a diagnosis, when definitive medical treatment would not start until at least 8 years of age.

Given the historical lack of consensus on which biomarkers are most effective at identifying HeFH, our group developed and validated assays for three analytes (TC, LDL-C, and ApoB) in DBS. The analytical validation studies demonstrated precision results within acceptable limits (CV < 15%) for all three screening assays. The established limit of linearity and LOQ of the three biomarkers exceeded the expected thresholds used clinically to distinguish the unaffected from the affected population. Biomarker concentrations in controls stored at room temperature for 10 days were maintained for LDL-C and TC, with slight degradation observed for ApoB. A direct comparison of ApoB and LDL-C measured in serum and DBS specimens obtained concurrently from research participants demonstrated a positive correlation, with only a slightly reduced concentration in DBS, consistent with the recovery studies. For TC, the values in DBS were higher than serum values, consistent with the presence of TC in red blood cells [24]. An initial evaluation of the three biomarkers in the presumptively unaffected population yielded concentrations similar to the limited published reports of TC, LDL-C, and ApoB measured in newborn DBS specimens [21] or in cord blood [14,15,16,17,18,19,20]. All three assays were performed using instrumentation already available within the newborn screening laboratory, and the cost of reagents was minimal (less than USD 3 per test).

A complete clinical validation, to include a more extensive evaluation of TC, LDL-C, and ApoB concentrations within the affected and unaffected newborn population, is underway. The influence of cofactors on the biomarker concentrations will be evaluated, specifically for changes due to sex, birth weight, gestational age, and age at specimen collection [21,25,29,30]. Additionally, it is possible that feeding status, such as breast milk, formula, or total parenteral nutrition may impact biomarker levels. In population-wide screening, it is not uncommon to obtain a biochemical phenotype similar to the disease of interest, but due to physiological or environmental factors (false-positives) or the presence of another genetic disorder. Our research group is planning for a second-tier molecular assessment of key genes associated with familial hypercholesterolemia to be performed in specimens with elevated biomarkers above the normal distribution. It is estimated that 85–90% of HeFH cases are monogenic; therefore, development of a comprehensive gene panel to be used on DBS specimens would enable confirmation of the disease state [31].

At present, the sensitivity and specificity of these biomarkers to detect HeFH in the newborn period is unknown. It is possible that one biomarker may be superior to the other two. It is also plausible that none of the markers are sufficient to diagnosis all cases of HeFH, and milder forms may remain undetected. However, as estimated by Wald et al., even detection of only 80% of affected individuals through a screening program would have a tremendous public health impact [17]. Prospective population studies followed by clinical evaluation would correlate the newborn screening results with phenotypes later in childhood, the efficacy of cascade screening for at-risk relatives, and clinical outcomes for the whole family. This would permit an unprecedented opportunity to effectively diagnose and treat a common and potentially fatal genetic disease, improving the cardiovascular health of these individuals and their families.

## 5. Conclusions

In conclusion, this paper describes the validation of high-throughput assays to quantify the familial hypercholesterolemia biomarkers TC, LDL-C, and ApoB in DBS. This is an important first step for the inclusion of HeFH and HoFH in the NBS panel of diseases.

## Figures and Tables

**Table 1:** Assay precision and recovery of total cholesterol, low-density lipoprotein-cholesterol, and apolipoprotein B from dried blood spot controls. CV, coefficient of variation; N, total number of evaluations.

Total Cholesterol			
	Ctrl 1	Ctrl 2	Ctrl 3
Mean Concentration (mg/dL)	177.2	308.5	422.9
Standard Deviation	15.7	20.6	36.0
% CV	8.9	6.7	8.5
Expected	198.0	378.0	559.0
% Recovery	89.5	81.6	75.7
N	25	25	25
Low-Density Lipoprotein-Cholesterol			
	Ctrl 1	Ctrl 2	Ctrl 3
Mean Concentration (mg/dL)	68.2	145.2	253.5
Standard Deviation	9.7	11.4	21.2
% CV	14.2	7.9	8.4
Expected	76.0	196.0	326.0
% Recovery	89.7	74.1	77.8
N	25	25	25
Apolipoprotein B			
	Ctrl 1	Ctrl 2	Ctrl 3
Mean Concentration (mg/dL)	11.1	24.4	62.2
Standard Deviation	0.5	1.6	4.8
% CV	4.5	6.7	7.8
Expected	12.0	30.0	60.0
% Recovery	92.6	81.4	103.7
N	25	25	25

**Table 2:** Limits of detection and quantification, and linearity of total cholesterol, low-density lipoprotein-cholesterol, and apolipoprotein B in dried blood spots.

	Limit of Detection	Limit of Quantification	Limit of Linearity
Total Cholesterol	NA	100 mg/dL	900 mg/dL
Low-Density Lipoprotein-Cholesterol	20 mg/dL	40 mg/dL	387 mg/dL
Apolipoprotein B	2.5 mg/dL	5 mg/dL	150 mg/dL
